# Supplementary material for: KSHV Topologically Associating Domains in Latent and Reactivated Viral Chromatin
Source: J Virol. 2022 Jul 11;96(14):e00565-22. doi: 10.1128/jvi.00565-22 (PMC9327698; doi:10.1128/jvi.00565-22)
Supplement: Supplemental file 3 — Supplemental material. Download jvi.00565-22-s0003.pdf, PDF file, 7.6 MB [file jvi.00565-22-s0003.pdf]

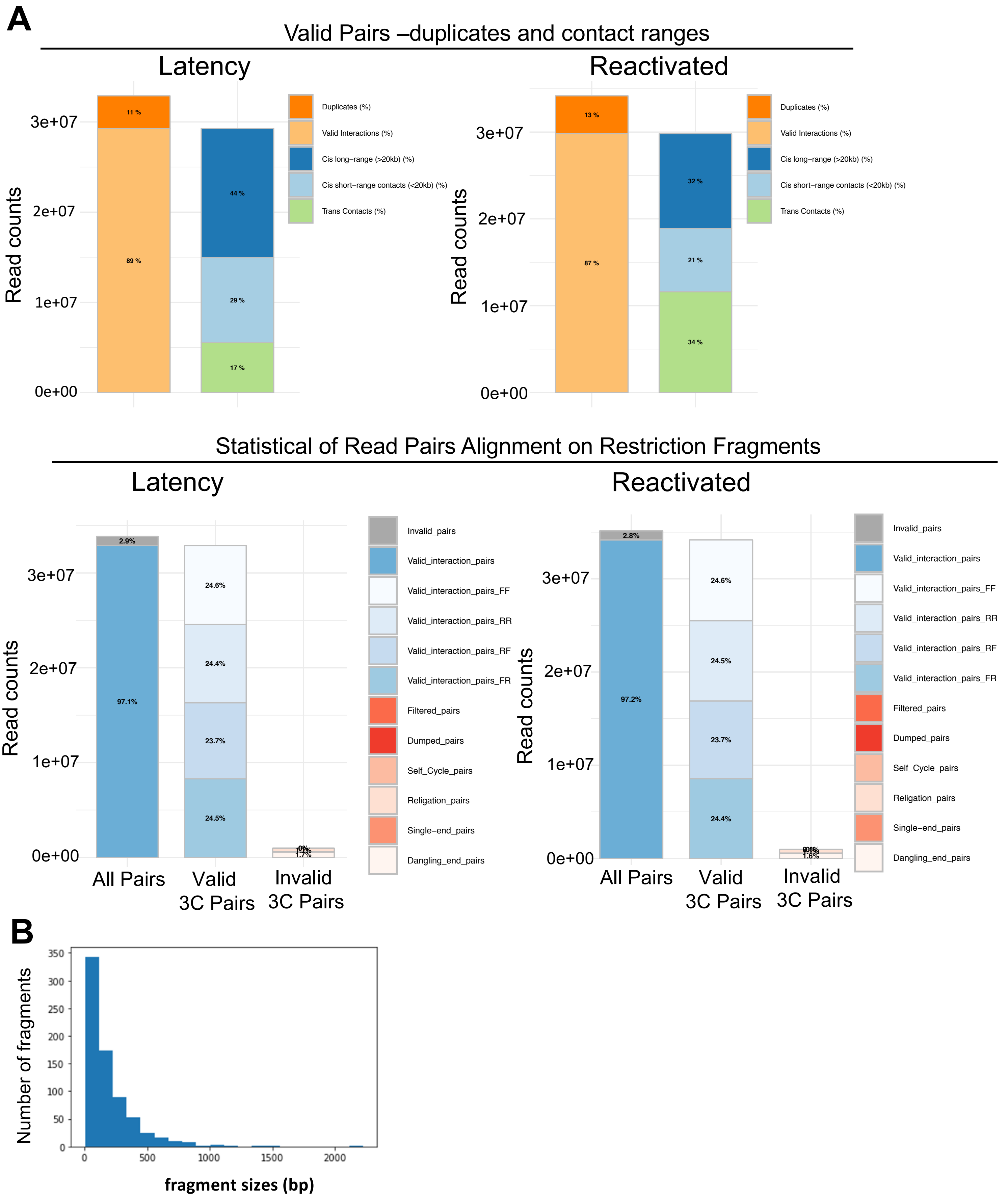

Supplemental Figure S1. (A) Representative valid sequence pairs in C-HiC. Detail sequence pairs with valid and invalid sequences were summarized in bar charts. One of three replicates of BCBL-1 samples for both before and after triggering reactivation are shown. **(B)** Predicted size distribution of KSHV genomic restriction fragments generated during CHi-C library preparation.

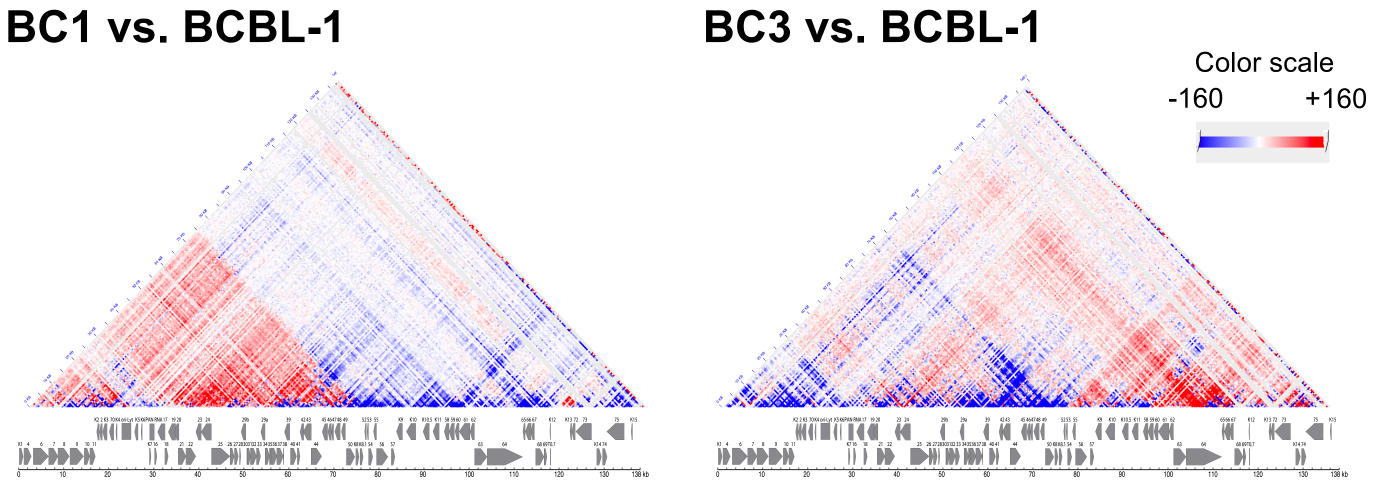

Supplemental Figure S2. Differences in genomic interaction frequencies among PEL cell lines. Relative Hi-C pair reads were directly compared between BC1 and BCBL-1 (left panel) or between BC3 and BCBL-1 (right panel). Presence of more genomic contacts than BCBL-1 is shown in red, while less contact is shown in dark blue. The results indicate overall genomic structure are similar among cell lines (Figure 1), genomic loop frequencies within genomic domains are different among cell lines tested.

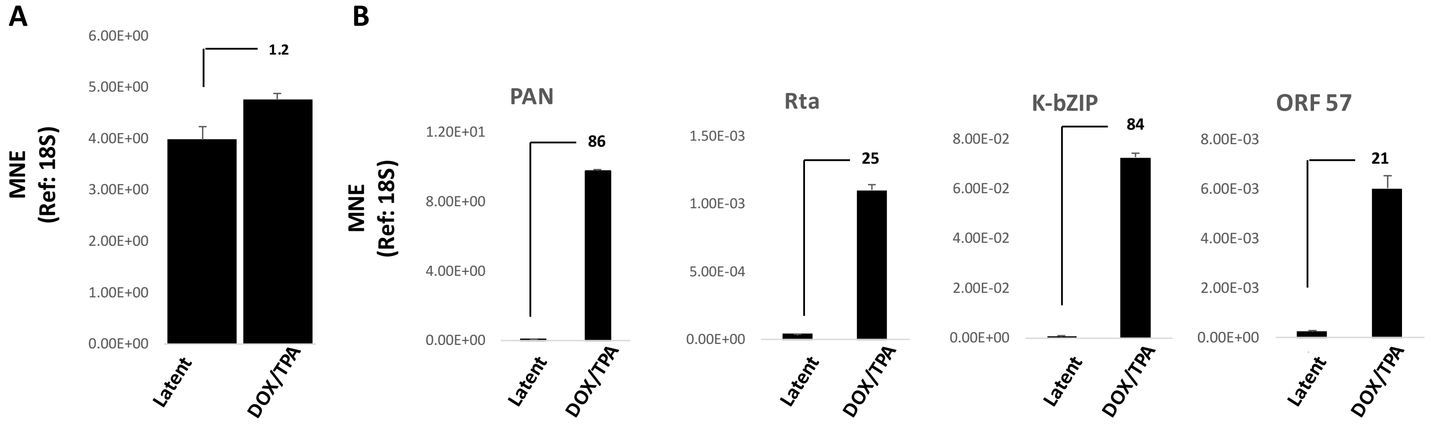

Supplemental Figure 3. Viral reactivation at 24hr. BCBL-1 TREx-F3-H3 K-Rta cells were treated -/+ DOX/TPA (1µg/ml DOX + 20ng/ml TPA, 4-hour pulse, with wash-out) and cellular DNA and RNA was harvested at 24h. **(A)** Intracellular vDNA, total cellular DNA was prepared using the Qiagen mini kit and 250 ng DNA/well and a LANA primer pair was used for qPCR. **(B)** Viral gene expression, total RNA was isolated using the Zymo mini kit. 2 µg total RNA was reverse-transcribed and used for qPCR using the indicated primer pairs. The Rta primer pair detects both DOX-inducible and viral Rta transcripts. Values represent mean -/+ SD of triplicate determinations normalized to the 18S reference signal. Fold change (reactivated/latent) is listed above each induced column.

pAG-MNase protein sequence

MGTMTMITPSLKDDPSQSANLLSEAKKLNESQA
PKADNKFNKEQQNAFYEILHLPNLNEEQRNGFI
QSLKDDPSQSANLLAEAKKLNDAQAPKADNKFN
KEQQNAFYEILHLPNLTEEQRNGFIQSLKDDPSVS
KEILAEAKKLNDAQAPKTTYKLVINGKTLKGETTT
EAVDAETAERHFKQYANDNGVDGEWTYDDATK
TFTVTEKPEVIDASELTPAVDDDKEFAGGGGSGG
GGSGGGGSTSTKKLHKEPATLIKAIDGDTVKLMY
KGQPMTFRLLLVDTPETKHPKKGVEKYGPEASAF
TKKMVENAKKIEVEFDKGQRTDKYGRGLAYIYAD
GKMVNEALVRQGLAKVAYVYKPNNTHEQHLRK
SEAQAKKEKLNIWSEDNADSGQGGGGSGGGGS
GGGGS**HHHHHH**GGGGS**DYKDDDDK**

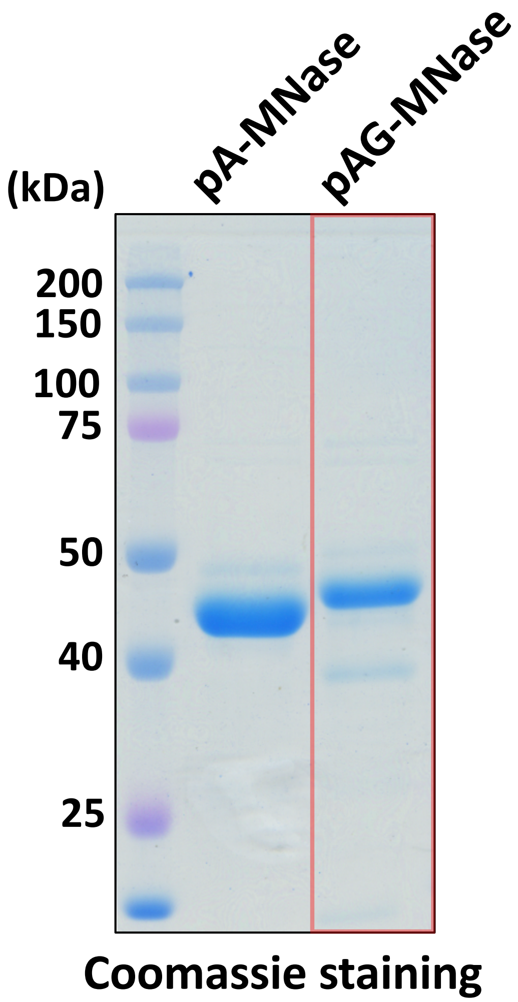

Supplemental Figure 4. Preparation of pAG-Mnase for CUT&RUN. Protein A or A&G domain fused MNase (pAG-MNAse) was expressed in E.coli, and purified with Ni-NTA beads (Invitrogen). Purified proteins were subjected to SDS-PAGE and stained with commassie. Recombinant protein sequence and purified protein are shown. The pAG-MNase was used for CUT&RUN studies described in this manuscript.

Supplemental Movie Legends.

KSHV 3D genomic structure models for both (1) latency and (2) reactivated samples are shown. Gray shadow indicates each model and color string 5' (blue) to 3' (red) are generated based on average of 10 different models. Blue ball (PAN RNA region), Red ball (K-Rta promoter region), and Yellow ball (K12 genomic region) were marked for clarity
